# The effect of high-dose Levothyroxine on hearing in patients operated for thyroid cancer

**DOI:** 10.1007/s00405-024-08857-w

**Published:** 2024-07-31

**Authors:** Vahit Mutlu, Zülküf Kaya, Arzu Bilen, Ramazan Dayanan

**Affiliations:** 1 Department of Otorhinolarygology, Medical Faculty of Ataturk University, Erzurum, 25240 Turkey; 2 Department of Internal Medicine, Medical Faculty of Ataturk University, Erzurum, Turkey; 3 Division of Endocrinology and Metabolism, Department of Internal Medicine, Medical Faculty of Ataturk University, Erzurum, Turkey

**Keywords:** Hearing loss, High dose, Levothyroxine, Osteopenia, Osteoporosis, Thyroid cancer, Thyroidectomy

## Abstract

**Objective:**

The aim of this study is to investigate the relationship between the use of high doses of levothyroxine (L-T4) and hearing loss in patients who have undergone surgery for thyroid cancer.

**Material method:**

After total thyroidectomy for thyroid cancer, patients were divided into two groups according to L-T4 dose below 150 µg and above 150 µg. Demographic characteristics, postoperative duration, radioactive iodine treatment, bone densitometry scans with LDxa and FDxa, and right and left ear hearing levels were statistically compared in both groups.

**Results:**

The study included 62 patients, 85.5% (*n* = 53) of whom were female, with a mean age of 48.8 ± 11.7 years. While 56.45% (*n* = 35) of the patients were taking L-T4 below 150 µg 43.55% (*n* = 27) were taking L-T4 above 150 µg. The mean postoperative duration of the participants was 4.1 ± 2.7 years, osteopenic 30.7% and osteoporotic 16.13% according to LDxa, osteopenic 29.0% and osteoporotic 1.6% according to FDxa. Hearing loss in both right and left ears was 41.9% and sensorineural hearing loss in both ears was 22.6%. Age, LDxa, FDxa, hearing loss in the right and left ear were found to be significantly different in the two groups above and below 150 µg according to the dose of L-T4 used (*p* < 0.05). However, no differences were found according to sex, height, weight, body mass index, postoperative period, or radioactive iodine treatment (*p* > 0.05). Both osteopenia and osteoporosis, as well as hearing loss in both the right and left ear, were significantly higher in the group taking L-T4 150 µg or more (*p* < 0.05).

**Conclusion:**

In our study, we found that patients taking 150 µg or more of L-T4 daily were more osteopenic and osteoporotic and had more hearing loss in both ears.

## Introduction

Current treatments for thyroid cancer include total thyroidectomy and radioactive iodine therapy, followed by supraphysiological dosages of levothyroxine (L-T4) [1].

Overt hyperthyroidism has long been identified as a risk factor for osteoporosis and poor bone mineral density (BMD) [2]. Because hyperthyroidism speeds up the turnover of bone and reduces the duration of the bone remodelling cycle. As a result, it was expected that suppressive therapy with L-T4 for thyroid cancer would lead to a decrease in BMD [1]. However, there are also studies in the literature concluding that suppressive doses of L-T4 therapy for postoperative thyroid cancer have no a negative effect on bone density [3].

The World Health Organisation (WHO) defines osteoporosis as “a systemic skeletal disease that includes low bone mass and microarchitectural deterioration, which leads to increased bone fragility and fracture predisposition” [4]. The most common fracture sites are the vertebral bodies, distal radius, and femoral neck [5].

Osteoporosis has been documented as a risk factor for hearing loss in several studies. The underlying cause of hearing loss in osteoporosis is complex and not well understood. Some studies have suggested that systemic demineralization of the skeleton, including the temporal bone, which includes the cochlea and the conductive chain, is a likely underlying mechanism of osteoporosis [6–8]. According to several researchers, cochlear demineralization is mostly connected with sensorineural hearing loss, and the degree of cochlear demineralization is directly proportional to the severity of hearing loss [6]. Hearing loss reduces quality of life by causing social isolation, depression, safety issues, reduced mobility, income, and employment opportunities [9].

The aim of the present study is to investigate the relationship between the use of high doses of levothyroxine (L-T4) and hearing loss in patients who have undergone surgery for thyroid cancer.

## Materials and methods

This cross-sectional study was conducted at a single center, Ataturk University Faculty of Medicine in Turkey. The study included 62 patients (ages 25 to 75) who underwent total.

thyroidectomy with or without radioactive iodine therapy due to thyroid carcinoma. We included subjects from October 2019 to October 2021. All subjects had received L-thyroxine treatment for different periods of time.

Demographic information and basic medical data were collected from all subjects who participated in the study. All patients underwent a detailed ear, nose, and throat examination. Patients with any ear pathology or disease were excluded from the study. Also excluded were any cases of previous ear surgery or ototoxic drug use, congenital hearing loss, previous long-term steroid use, malignant or benign brain neoplasm, and history of noise exposure, as well as patients with any systemic or chronic disease that could affect BMD results, such as chronic renal failure, liver or biliary disease, and hormonal disorders.

All subjects underwent a dual x-ray absorptiometry (DXA) scan (Discovery-W, Hologic Inc.). BMD (g/cm2) was measured at the lumbar spine (L1-L4) (LDXA), femoral neck (FDXA), and total hip. A T-score is an expression of an individual’s BMD in standard deviations generated from the manufacturer’s reference values derived from DXA measurements. The World Health Organisation (WHO) T-score criteria were used to diagnose of osteopenia or osteoporosis: a T-score of ≥-1 is considered normal BMD; osteopenia is diagnosed with a T-score of -2.5 < T-score < -1; and osteoporosis is diagnosed with a T-score of ≤-2.5. The study group was divided into osteoporotic, osteopenic, and normal subjects on the basis of their BMD scores.

Audiological assessment was performed by pure tone audiometry (PTA). Both air and bone pathways were tested separately by using a double-channel clinical audiometer (Maico MA 53^®^, Germany). PTA testing was performed on all subjects at 0.25, 0.5, 1, 2, 4, and 8 kHz. Conductive hearing loss (CHL) was diagnosed when the bone conduction threshold was normal but the air-bone gap was greater than 10 dBHL. Sensorineural hearing loss (SNHL) was defined as a bone conduction threshold greater than 25 dBHL with no air-bone gap. Mixed hearing loss (MHL) was defined as present if both the mean bone and air conduction thresholds were greater than 25 dBHL and the air-bone gap was greater than 10 dBHL. A normal hearing level at speech frequencies up to 4 kHz, but a bone conduction threshold greater than 25 dBHL at frequencies above 4 kHz, was defined as high-frequency sensorineural hearing loss (HFSNHL).

All patients were divided into two groups as those who used L-T4 drugs below 150 µg and above 150 µg daily after total thyroidectomy. The relationships between the development of osteoporosis, osteopenia and hearing loss in both groups were determined and analysed statistically. In addition, these parameters were compared with the age, sex, height, weight, body mass index, time elapsed after the operation and the number of radioactive iodine received and the relationship between them was analysed statistically.

The data obtained in the study were analysed with SPSS V27 (IBM^®^, USA). Categorical data were presented with frequency and percentage, and continuous variables were presented with mean and standard deviation. The normal distribution of numerical data was analysed by Shapiro-Wilk test. Mann-Whitney U test was used to compare two independent groups that were not normally distributed. Categorical variables were analysed by Chi-square test. In the study, *p* < 0.05 was taken for statistical significance.

The study was approved by the Medical Ethics Committee of Ataturk University Faculty of Medicine and written informed consent was obtained from all participants.

## Results

Of the 62 patients included in the study, 85.5% were female (*n* = 53), 14.5% were male (*n* = 9), and the mean age was 48.8 ± 11.7 years. The mean drug dose of the patients was 133.1 ± 46.8 µg; 56.45% (*n* = 35) were on L-T4 doses below 150 µg; and 43.55% (*n* = 27) were on L-T4 doses above 150 µg.

Demographic and clinical features of the subjects are shown in Table 1. Body mass index (BMI) was 29.95 ± 6.57, duration of thyroid surgery was 4.1 ± 2.7 years, osteopenic according to LDXA was 30.7% and osteoporotic was 16.13%, osteopenic according to FDXA was 29.0% and osteoporotic was 1.6%. Hearing loss in both right and left ears was 41.9%, and sensorineural hearing loss in both ears was 22.6%.

**Table 1:** Demographic and clinical characteristics of the participants included in the study

		*N*	%
Group	150 µg Below	35	56,45
	150 µg Above	27	43,55
	Total	62	100,00
Gender	Male	9	14,52
	Female	53	85,48
	Total	62	100,00
Age	Mean		48,74
	Median		46,00
	Std. Deviation		11,65
	Range		53,00
	Minimum		22,00
	Maximum		75,00
Height	Mean		156,87
	Median		155,00
	Std. Deviation		9,61
	Range		43,00
	Minimum		140,00
	Maximum		183,00
Weight	Mean		73,23
	Median		74,00
	Std. Deviation		15,05
	Range		83,00
	Minimum		44,00
	Maximum		127,00
BMI	Mean		29,95
	Median		28,97
	Std. Deviation		6,57
	Range		28,01
	Minimum		18,08
Drug (µg)	Mean		133,06
	Median		125,00
	Std. Deviation		46,78
	Range		200,00
	Minimum		100,00
	Maximum		300,00
Time after the operation	Mean		4,10
	Median		4,00
	Std. Deviation		2,70
	Range		13,00
	Minimum		1,00
	Maximum		14,00
RAI Treatment	0	35	56,45
	1	24	38,71
	2	3	4,84
LDXA	Normal	33	53,23
	Osteopenia	19	30,65
	Osteoporosis	10	16,13
FDXA	Normal	43	69,35
	Osteopenia	18	29,03
	Osteoporosis	1	1,61
Hearing Right	Normal	36	58,06
	Conductive Hearing Loss (CHL)	4	6,45
	Sensorineural Hearing Loss (SNHL)	14	22,58
	Sensorineural Hearing Loss at High Frequencies (HFSNHL)	8	12,90
Hearing Left	Normal	36	58,06
	Conductive Hearing Loss (CHL)	5	8,06
	Sensorineural Hearing Loss (SNHL)	14	22,58
	Sensorineural Hearing Loss at High Frequencies (HFSNHL)	7	11,29

When two groups were formed as above and below 150 µg according to the dose of L-T4 drug use of the participants, the comparison of the patients according to demographic and clinical characteristics is presented in Table 2. Accordingly, significant differences were found between the groups in terms of age, LDXA, FDXA, right ear, and left ear hearing loss (*p* < 0.05). However, there was no significant difference between the groups in terms of gender, height, weight, BMI, time after the operation, or number of radioactive iodine treatments (*p* > 0.05).

**Table 2:** Comparison of the characteristics of the patients according to the study groups with daily L-T4 dosage above or below 150 µg

	*P*
Gender	0,485 *
Age	**0**,**003** †
Height	0,463 †
Weight	0,233 †
BMI	0,310 †
Drug (µg)	**< 0**,**001** †
Time after the operation	0,624 †
RAI Treatment	0,646 ‡
LDXA Group	**0**,**002** ‡
FDXA Group	**0**,**001** ‡
Hearing Right	**< 0**,**001** ‡
Hearing Left	**< 0**,**001** ‡

* Fisher’s Exact Test

† Mann–Whitney U test

‡ Pearson Chi-Square

Intergroup comparisons of the statistically significant parameters are presented in Table 3. Accordingly, the mean age of L-T4 users below 150 µg was 45.1 ± 10.3 years, whereas the mean age of those who used more than 150 µg was 53.5 ± 11.7 years. According to LDXA, there was a statistically significant increase in the number of osteopenic and osteoporotic patients in the group that used more than 150 µg. According to FDXA, osteopenia was significantly higher in the group using drugs above 150 µg. In terms of hearing loss in the right ear, SNHL and HFSNHL were significantly higher in patients taking more than 150 µg. In terms of hearing loss in the left ear, SNHL, HFSNHL, and CHL were significantly higher in users of drugs above 150 µg.

**Table 3:** Comparison of statistically significant variables in groups using daily L-T4 dosage above or below 150 µg

		Below 150 µg L-T4	Above 150 µg L-T4
Age	Mean	45,06	53,52
	Median	44,00	52,00
	Std. Deviation	10,30	11,74
	Range	51,00	49,00
	Minimum	22,00	26,00
	Maximum	73,00	75,00
LDXA	Normal	25	8
	Osteopenia	8	11
	Osteoporosis	2	8
FDXA	Normal	30	13
	Osteopenia	5	13
	Osteoporosis	0	1
Hearing Right	Normal	28	8
	Conductive Hearing Loss (CHL)	2	2
	Sensorineural Hearing Loss (SNHL)	3	11
	Sensorineural Hearing Loss at High Frequencies (HFSNHL)	2	6
Hearing Left	Normal	28	8
	Conductive Hearing Loss (CHL)	1	4
	Sensorineural Hearing Loss (SNHL)	5	9
	Sensorineural Hearing Loss at High Frequencies (HFSNHL)	1	6
Total		35	27

## Discussion

This study found that the use of high doses of levothyroxine (L-T4) increased the incidence of osteopenia and osteoporosis and caused hearing loss.

After total thyroidectomy, patients with thyroid cancer are given supraphysiological dosages of levothyroxine to prevent tumour recurrence and metastasis. The effect of high-dose L-T4 on bone metabolism, on the other hand, is debatable. A study reported that long-term treatment with L-T4 after total or subtotal thyroidectomy was not associated with bone health in patients with thyroid cancer [1]. Several in vitro studies have found that elevated thyroid hormone causes bone loss [10, 11]. In addition, some studies have shown that hyperthyroidism is a significant risk factor for osteoporosis, osteopenia, and osteoporotic fractures [12, 13].

Osteoporosis is a systemic disease that affects multiple bones. This means that demineralization is not limited to the long bones but can also affect the temporal bone and the structures within it. There have been several studies looking for an association between osteoporosis and hearing loss in the literature. These have found different results, including sensorineural hearing loss (SNHL), sudden SNHL, higher frequencies sensorineural hearing loss (HFSNHL), conductive hearing loss (CHL) and no significant association [5, 8, 14].

A decrease in BMD or osteoporosis was reported to be significantly associated with hearing loss in one study. It has been reported that an imbalance between bone formation and bone resorption in osteoporosis can cause ion metabolism disorders leading to sensorineural hearing loss [9]. The other study found that, as compared to normal and osteopenic participants, osteoporotic patients had a much higher incidence of SNHL, often affecting the mid and high frequencies. However, this study did not find a significant difference in order to hearing thresholds between normal and osteopenic participants [5]. Another study was found that at high frequencies in the mean air conduction thresholds were higher in the osteoporosis group than in the osteopenia and normal groups, but there was no difference in the mean bone conduction thresholds between the osteopenia and normal groups. However, there was no difference in mean bone conduction thresholds between the three groups [14].

In our study, high L-T4 doses above 150 µg daily were found to increase the incidence of both osteopenia and osteoporosis. In addition, high daily doses of L-T4 were found to cause more hearing loss than low doses. The majority of hearing impairments are of the SNHL and HFSNHL types and are usually bilateral. CHL was also found to be more common in high dose L-T4 users than in low dose users. All three hearing losses were found more common in both osteoporosis and osteopenia.

The present study is limited by a relatively small sample size and the fact that all patients were registered at a single centre. In addition, we were not able to assess daily calcium and vitamin D intake or physical activity, both of which are known to influence osteoporosis and osteopenia. In addition, the mean age of patients who took more than 150 µg of L-T4 daily was slightly higher than that of patients who took less than 150 µg of L-T4 daily. Because of all these limitations, the study needs to be supported by larger series and prospective studies conducted in different centres.

In conclusion, healthcare professionals should be aware that high-doses of L-T4 (≥ 150 µg /day) given to patients after total thyroidectomy for thyroid cancer can cause the side effects such as osteopenia, osteoporosis and even hearing loss, which can have a very negative impact on life, and patients should be closely monitored for these side effects.
